# Achieving Diamond‐Like Wear in Ta‐Rich Metallic Glasses

**DOI:** 10.1002/advs.202301053

**Published:** 2023-05-21

**Authors:** Fucheng Li, Mingxing Li, Liwei Hu, Jiashu Cao, Chao Wang, Yitao Sun, Weihua Wang, Yanhui Liu

**Affiliations:** ^1^ Institute of Physics Chinese Academy of Sciences Beijing 100190 China; ^2^ Songshan Lake Materials Laboratory Dongguan 523808 China; ^3^ Central of Materials Science and Optoelectronics Engineering University of Chinese Academy of Sciences Beijing 100049 China

**Keywords:** friction, metallic glasses, nanoindentation, wear

## Abstract

Most metals and alloys suffer from high friction and wear due to their low hardness and lack of self‐lubrication. Although plenty of strategies have been proposed, it is still a long‐standing challenge to achieve diamond‐like wear in metals. Metallic glasses (MGs) are supposed to possess low coefficient of friction (COF) because of their high hardness and fast surface mobility. However, their wear rate is larger than that of diamond‐like materials. Here, this work reports the discovery of Ta‐rich MGs that exhibit diamond‐like wear. This work develops an indentation approach for high‐throughput characterization of crack resistance. By employing deep indentation loading, this work is able to efficiently identify the alloys that exhibit better plasticity and crack resistance according to the differences of indent morphology. With high temperature stability, high hardness, improved plasticity, and crack resistance, the discovered Ta‐based MGs exhibit diamond‐like tribological properties, featured by COF as low as ≈0.05 for diamond ball test and ≈0.15 for steel ball test, and specific wear rate of only ≈10^−7^ mm^3^ N^−^^1^m^−1^. The discovery approach and the discovered MGs exemplifie the promise to substantially reduce friction and wear of metals and may unleash the potential of MGs in tribological applications.

## Graphical abstract

A high‐throughput characterization approach is developed for efficient identification of the metallic glasses (MGs) that exhibit better plasticity and crack resistance. With the approach, this work discovers Ta‐rich MGs that exhibit diamond‐like tribological properties due to the combination of high structural stability, high hardness, improved plasticity, and crack resistance.

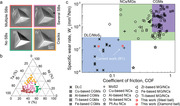

## 1 Introduction

Reducing coefficient of friction and wear rate of materials have been an unremitting pursuit for its importance in diverse areas ranging from transportation, manufacturing, energy generation, medical treatment to space exploration.^[^ ^1^, ^2^, ^3^, ^4^ ^]^ Among the materials developed in the past decades,^[^ ^2^, ^3^, ^4^, ^5^, ^6^, ^7^, ^8^, ^9^, ^10^, ^11^, ^12^ ^]^ carbon‐based materials (e.g., graphite, diamond‐like carbon (DLC), and nanocrystalline (NC) diamond) exhibit significantly low friction and wear rate.^[^ ^9^, ^10^, ^11^, ^12^, ^13^ ^]^ For example, the specific wear rate of DLC coatings can be as low as ≈10^−9^ mm^3^ N^‐^^1^m^−1^, while their coefficients of friction are mainly in the range of 0.1–0.2 in ambient air and even smaller than 0.1 in high vacuum, water‐ or oil‐lubricated conditions.^[^ ^9^, ^10^, ^11^ ^]^ These attributes make carbon‐based materials among the most promising candidates for tribological applications. However, their superior tribological properties are sensitive to humidity, oxygen, temperature, and structural orientation, rendering their applications limited to surface coatings or fillers in self‐lubricating composites.^[^ ^9^, ^10^, ^11^ ^]^

Metals are the most commonly used industrial materials due to their combination of physical and mechanical properties. However, the tribological properties of most conventional metals are generally inferior to that of carbon‐based materials under similar tribological system and testing conditions.^[^ ^10^, ^11^, ^12^, ^13^, ^14^, ^15^, ^16^, ^17^ ^]^ For instance, COFs of Ti‐6Al‐4V alloy and various steels obtained under ball‐on‐disk tests are in the range of 0.5–1.0 in ambient air,^[^ ^14^, ^15^, ^16^, ^17^ ^]^ which are substantially larger than that of carbon‐based materials. Their specific wear rates are often in the range of 10^−4^–10^−5^ mm^3^ N^−^^1^m^−1^,^[^ ^14^, ^15^, ^16^, ^17^ ^]^ which is 2–4 order of magnitude higher than that of carbon‐based materials. Despite the great efforts devoted in the past, it remains challenging to achieve diamond‐like tribological properties in metals.

According to classical theory,^[^ ^18^, ^19^ ^]^ both friction and wear of metals are proportional to their hardness. This makes NC and amorphous alloys attractive for their ultrahigh hardness compared with conventional metals.^[^ ^20^, ^21^ ^]^ For examples, Ni–W NC alloys show low specific wear rates of ≈10^−6^ mm^3^ N^‐^^1^m^−1^, regardless of their high COFs of 0.6–0.7.^[^ ^22^ ^]^ A Cu‐Ag gradient NC alloy exhibits a low specific wear rate of ≈10^−6^ mm^3^ N^‐^^1^m^−1^ and a low COF of 0.29.^[^ ^23^ ^]^ By mitigating fatigue‐driven delamination wear, the specific wear rate of a Pt‐Cu NC alloys is further decreased to ≈10^−7^ mm^3^ N^‐^^1^m^−1^ against Si_3_N_4_ ball or even ≈10^−9^ mm^3^ N^‐^^1^m^−1^ against Al_2_O_3_ ball.^[^ ^24^ ^]^ In addition to high strength, the surfaces of metallic glasses (MGs) show liquid‐like behavior with atomic mobility much faster than that of their crystalline counterparts.^[^ ^25^, ^26^ ^]^ Cao et al.^[^ ^27^ ^]^ measured the surface viscosity and self‐diffusion of a Pd‐based MG and found that the surface diffusion is 10^5^ times faster than bulk diffusion at temperature below glass transition. This bestows MGs self‐lubrication from the surface layers and ultralow COFs, which is commonly observed in DLC coatings due to the formation of easy‐shear graphitic layers.^[^ ^9^, ^28^ ^]^ For example, Lu et al.^[^ ^29^ ^]^ reported the surface mobility induced self‐lubrication in various MGs and achieved substantial reduction of COFs when the scratch depth is on nanoscale. Indeed, in dry sliding condition, COFs of several MGs have been reported to be less than 0.2, which is comparable to diamond‐like materials.^[^ ^5^ ^]^ However, the specific wear rates of most MGs are larger than ≈10^−6^ mm^3^ N^‐^^1^m^−1^.^[^ ^5^, ^30^ ^]^ This can be attributed to the relaxation or crystallization of MGs upon wearing, which results in cracks that lead to high wear rates.^[^ ^5^, ^30^, ^31^ ^]^ To substantially reduce specific wear rate and unleash the potential of MGs in tribological applications, a MG should possess both high structural stability and superior mechanical properties. Recently, high temperature MGs attract extensive attentions due to their combination of outstanding thermal stability, mechanical, and functional properties.^[^ ^32^ ^]^ The high glass transition temperature (*T*_g_) larger than 1000 K makes them resistant to structural relaxation and crystallization at ambient temperature.^[^ ^32^ ^]^ Therefore, it is promising to discover wear‐resistance metals in the high temperature MGs. However, the crack resistance of MGs is strongly related to their compositions. Characterization of crack resistance for various MGs is a daunting task.

In this work, we demonstrate a group of Ta‐rich MGs that show diamond‐like wear. We developed high‐throughput mechanical characterization based on deep indentation that can trigger shear banding or cracking. The differences in postindention morphology allow identification of the alloys that exhibit improved plasticity and crack resistance. This method helps us to rapidly discover MGs with potentially better tribological properties within the Ta–Ni–Ir combinatorial alloy library. The discovered Ta‐rich MGs show diamond‐like wear with an ultralow COF (≈0.05 for diamond ball and ≈0.15 for steel ball) and specific wear rate of ≈10^−7^ mm^3^ Nm^−1^ due to the combination of mechanical properties and structural stability.

## 2 Results and Discussion

### 2.1 Preparation and Characterization of Ta‐Ni‐Ir Combinatorial Alloy Library

**Figure 1:**
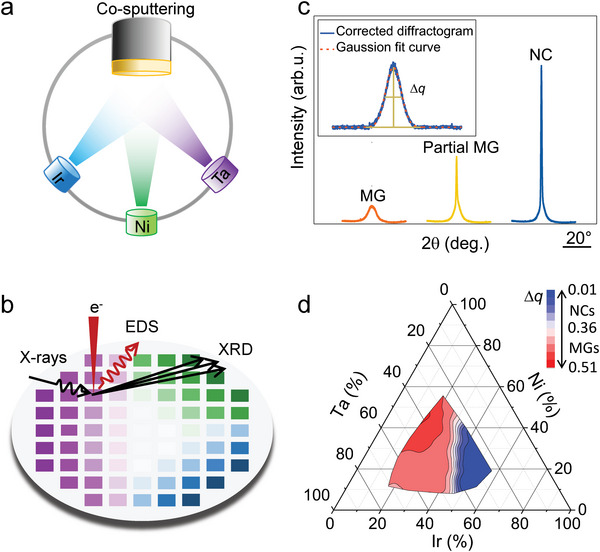
Combinatorial syntheses and high‐throughput chemical and structural characterizations. a) Illustration for the magnetron cosputtering for combinatorial syntheses of the Ta–Ni–Ir alloys. b) Illustration for the compositional and structural characterization with automated energy‐dispersive X‐ray spectroscopy (EDX) and X‐ray diffraction (XRD) methods. c) The typical XRD patterns of metallic glasses (MGs), partial MGs, and nanocrystalline (NC) alloys. The inset shows the full‐width at the half‐maximum (Δ*q*) of the first diffraction peak. d) Variation of Δ*q* within the Ta–Ni–Ir combinatorial alloy library.

To identify MGs of desired tribological properties, we explore the Ta–Ni–Ir alloy system because of its potential to form bulk MGs with high *T*_g_.^[^ ^32^ ^]^ In addition, Ir–Ta binary MGs show the highest crystallization temperature (*T*_x_ = 1283K).^[^ ^33^ ^]^ The high *T*_x_ and *T*_g_ is beneficial to suppressing possible relaxation and crystallization at ambient temperature. We create combinatorial alloy library via magnetron cosputtering (**Figure** **1**a) around the composition of Ta_40_Ir_35_Ni_25_ (at.%) that exhibits strong glass forming ability.^[^ ^32^ ^]^ Automated energy dispersive spectroscopy (EDS) and X‐ray diffraction (XRD) allow us to reveal the composition distribution of the combinatorial alloy library and identify the structures of each alloys within the library (Figure 1b), in particular, the compositional range of glass forming alloys. The full‐width at the half‐maximum (Δ*q*) of the first diffraction peak in the XRD patterns (Figure 1c) is used as an indicator to reflect the glass forming tendency.^[^ ^34^ ^]^ As shown in Figure 1d, the alloy library covers a broad compositional range (20–70% Ta, 10–60% Ir, and 10–60% Ni). According to the characteristic of XRD patterns and the values of Δ*q* (see Figure 1c and Figure S1a–c, Supporting Information), the Ta–Ni–Ir alloys within the library can be divided into three regions, i.e., glass forming region (Δ*q* > 0.35), partial glass forming region (0.08 > Δ*q* > 0.35), and NC region (Δ*q* < 0.08).

### 2.2 High Throughput Mechanical Characterization of Ta‐Ni‐Ir Combinatorial Alloy Library

**Figure 2:**
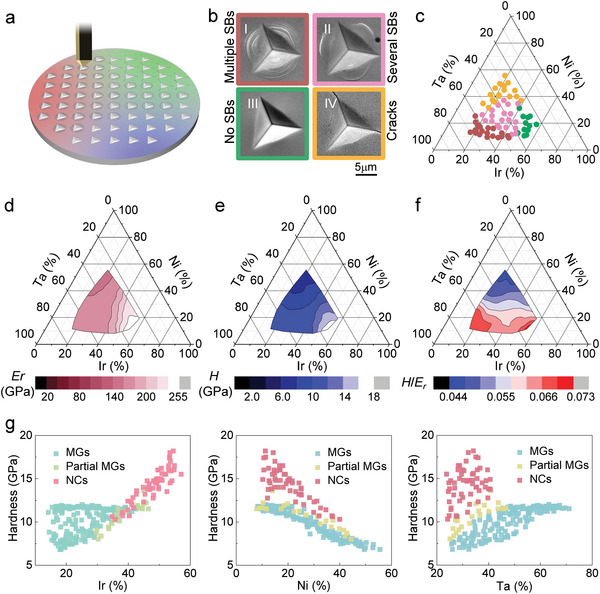
The combinatorial characterizations of mechanical properties. a) Schematic diagram illustrating method to characterize plasticity and crack resistance. b) Typical indent morphologies arising from (I) multiple shear bands, (II) a few shear bands, (III) no shear bands, and (IV) cracks formation. c) Summary of the types of indent morphologies within the Ta–Ni–Ir alloy library. d) The distribution of reduced modulus within the Ta–Ni–Ir alloy library. e) The distribution of hardness within the Ta–Ni–Ir alloy library. f) The variation of hardness with Ir. g) The variation of hardness with Ni. i) The variation of hardness with Ta.

As tribological properties of metals are closely related to hardness, plasticity, and crack resistance,^[^ ^18^, ^35^, ^36^ ^]^ the mechanical properties of the combinatorial alloy library need to be characterized. Although hardness of the combinatorial alloy library can be readily measured by nanoindentation,^[^ ^37^, ^38^ ^]^ evaluation of plasticity and crack resistance is challenging. To reveal the relative plasticity and crack resistance of the alloys within the library, a quick and effective characterization approach is needed. It is known that deformation of MGs proceeds by formation and propagation of shear bands. The plasticity of MGs is closely related to the number of shear bands formed during deformation. Formation of multiple shear bands typically improves the plasticity of MGs.^[^ ^39^, ^40^ ^]^ During indentation, deformation of MGs occurs by the pile‐up of materials against the faces of the indenter, leading to the appearance of overlapping layers of upwardly displaced materials due to the formation of shear bands.^[^ ^41^, ^42^ ^]^ For brittle materials, cracks at the corner or edges of the indent can be observed.^[^ ^43^, ^44^, ^45^ ^]^ This inspires us to use nanoindentation as a tool for high‐throughput evaluation of plasticity and crack resistance (**Figure** **2**a). We employ deep loading with penetration depth of 2 µm, so that the sharp diamond indenter penetrates deeply into the tested materials and induces severe plastic deformation. By observing the features around indents across the combinatorial alloy library, compositional regions that exhibit different deformation behaviors can be qualitatively distinguished (Figure 2b,c). It was observed that in Ni‐rich region, the indent morphologies are featured by crack formation at the corners of indent (IV in Figure 2b). These indicate that the alloys are brittle and less resistant to cracking. In the central region of the library, shear bands appear around the indent without any cracks (II in Figure 2b). This suggests that the alloys are more crack‐resistant than the alloys within the Ni‐rich region. However, the number of shear bands is limited, i.e., only one or two shear bands can be observed at each side of the indent. In contract, profuse shear bands can be observed around indent in Ta‐rich region (I in Figure 2b). This is a strong indication that the MGs within this region possess substantially better plasticity than that within the above mentioned two regions. Within the Ir‐rich region, neither shear bands nor cracks can be observed (III in Figure 2b). Since the majority of alloys within this region is NC (Figure 1d), the absence of shear bands may arise from their different deformation mechanism from MGs. Similar phenomenon has also been observed in NC Ta^[^ ^46^ ^]^ and polycrystalline Al films^[^ ^47^ ^]^ with their plastic deformation controlled by twinning and dislocation formation, respectively. For Cu single crystals,^[^ ^48^ ^]^ however, pile‐up patterns can be observed after indentation with a conical indenter. The pile‐up or sink‐in patterns may also be used as the indicator of plasticity in crystalline metals, which needs further study.

In addition to the quantification of plasticity and crack‐resistance, the nanoindentation can also be used to reveal the distribution of hardness and elastic modulus within the combinatorial alloy library.^[^ ^37^, ^38^ ^]^ Figure 2d,e presents the variation of reduced Young's modulus *E*_r_ and hardness *H* with composition. One can see that both hardness and reduced Young's modulus of the Ir‐rich region are higher than that of Ta‐rich and Ni‐rich regions. This can be attributed to the compositional effect and the formation of NC structures. Within the glass forming region, the concentration of Ta appears to play a minor role on the variation of *H* and *E*_r_. In contrast, they strongly rely on the concentration of Ir and Ni, i.e., increase in Ir and decrease in Ni lead to the increase of *H* and *E*_r_ (Figure 2g and Figure S1d–f, Supporting Information). Furthermore, the ratio between *H* and *E_r_* can also be obtained as shown in Figure 2f, which has been demonstrated to be strongly related to both fracture toughness and wear resistance.^[^ ^49^, ^50^, ^51^, ^52^ ^]^ It is interesting to note that the Ta‐rich region shows larger *H*/*E*_r_ values than other regions. This indicates that the alloys within the Ta‐rich region are more crack‐resistant and may exhibit remarkable wear resistance, consistent with the observation on indentation morphologies (Figure 2c).

According to the composition dependency of structure, hardness, and deformation morphology, the alloys within the combinatorial alloy library can be categorized into four regions. When the content of Ir exceeds 40%, the alloys show substantially high hardness (≈12 to 18 GPa) than other alloys, while neither significant pile‐ups nor cracks around the indents can be observed (R4). In the glass forming region with Ta > 46%, abundant shear bands form around indents, suggesting decent plasticity of the alloys (R1). In addition, the alloys within R1 also possess high hardness (≈11 to 12 GPa). However, when the content of Ni is above 30% in the glass forming region (R3), cracks occur around the indents and the values of hardness is also much lower (≈7 to 9 GPa). The central region around Ir_33_Ni_28_Ta_39_ includes both glassy alloys and partial glassy alloys. Although a few shear bands form around the indents, only moderate hardness (≈9 to 11 GPa) can be obtained with the alloys within this region (R2).

### 2.3 Verification of Superior Mechanical Properties of Selected Alloys

**Figure 3:**
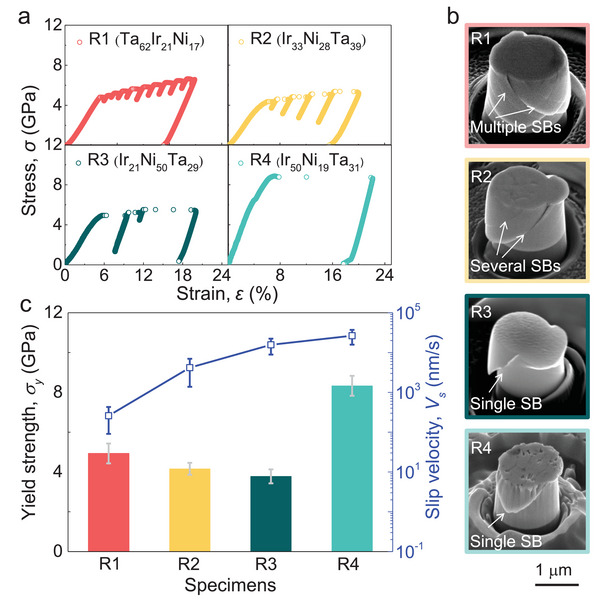
Microcompression tests on the Ta–Ni–Ir alloys. a) Typical stress–strain curves for the alloys. b) The postdeformation morphologies of the pillars for alloys R1 to R4. c) The yield strength and slip velocity of deformation bands for R1 to R4.

To verify the reliability of the initial screening by nanoindentation, we quantitatively test the mechanical behavior of the alloys from different regions. We select one alloy within each of the four regions, i.e., Ta_62_Ir_21_Ni_17_ (R1), Ta_39_Ir_33_Ni_28_ (R2), Ni_50_Ir_21_Ta_29_ (R3) and Ir_50_Ni_19_Ta_31_ (R4) (see Experimental Section and Figure S2, Supporting Information, for information about structures and surface morphologies). We prepared micropillars with diameter of ≈1.2 µm by focused ion beam (FIB) and compressed the micropillar to a strain of 20% using nanoindentation with a flat diamond indenter at a strain rate of 5 × 10^−3^. **Figure** **3**a shows the typical stress–strain curves for samples from R1 to R4. The postdeformation morphologies of the pillars are shown in Figure 3b. As can be seen, the four alloys exhibit distinctly different deformation behavior. For example, Ta_62_Ir_21_Ni_17_ MG (R1) shows a yield strength of ≈5 GPa, which is higher than most MGs.^[^ ^53^ ^]^ Moreover, small and frequent serrations can be seen from its stress–strain curve. The obvious serration behaviors originate from the activation of multiple shear bands (Figure 3b) which effectively avoids strain localization into a dominant shear band^[^ ^39^, ^54^, ^55^ ^]^ and thus results in substantial plastic deformation. In contrast, serrations are significantly less and large in the stress–strain curve of Ta_39_Ir_33_Ni_28_ (R2) and Ni_50_Ir_21_Ta_29_ (R3) MGs. Consistently, only a few shear bands can be observed from the micropillars after deformation. Since shear bands are the carriers of plastic strain for MGs, the less number of shear bands suggests that the Ta_39_Ir_33_Ni_28_ and Ni_50_Ir_21_Ta_29_ MGs are of worse plasticity than Ta_62_Ir_21_Ni_17_.^[^ ^54^, ^55^ ^]^ Although the crystalline Ir_50_Ni_19_Ta_31_ alloy shows much higher yield strength (≈8.3 GPa), the pillar fracture along a single slip band. The fracture is similar to the phenomenon observed in Fe‐based and Ni‐W NC alloys,^[^ ^54^, ^56^ ^]^ suggesting the poor plasticity of Ir_50_Ni_19_Ta_31_.

To further compare the deformability of the samples, we estimated the average slip velocity of shear band or slip band for all samples based on repeated microcompression tests. As shown in Figure 3c, the slip velocity progressively increases from R1 to R4, implying that they possess different plasticity,^[^ ^57^ ^]^ with R1 being the most ductile among them. For brittle MGs, strain localization along one major shear band is responsible for their stick‐slip instabilities and the internal state within the shear band determines the differences of sliding dynamics.^[^ ^58^ ^]^ The faster sliding motion of a shear band can be attributed to a creation of abundant excess free volumes in a narrower shear regions causing significant strain softening.^[^ ^58^ ^]^ These localized excess free volumes tend to coalescence and promote void formation, which is the origin of catastrophic fracture and poor plasticity.^[^ ^59^ ^]^ Within a sliding band of NC alloys, strain localization and softening can be caused by grain boundary sliding.^[^ ^56^, ^60^ ^]^ For ductile MGs, the interactions between multiple shear bands can significantly reduce their sliding motion, and thus improve the plasticity of MGs.^[^ ^61^ ^]^

**Figure 4:**
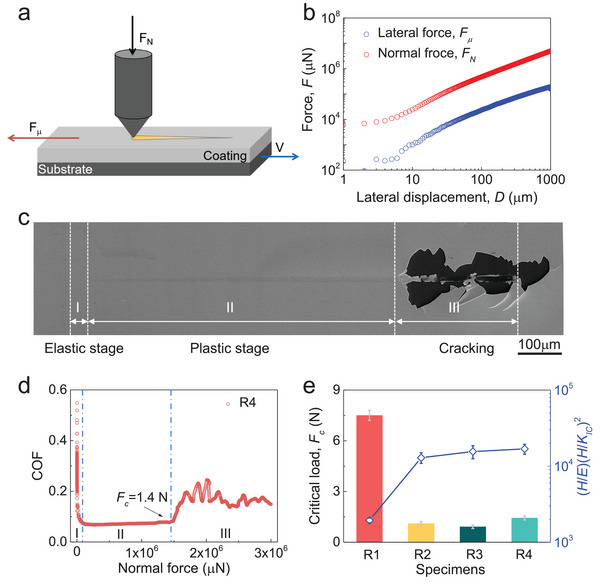
Nanoscrach tests on the Ta–Ni–Ir alloys. a) Illustration for nanoscrach tests through nanoindentation under ramp mode. b) The variation of lateral force with the increase of normal force at constant rate. c) A typical scratch trace demonstrating the change of deformation stages from elastic, plastic to fracture with the increase of the normal force. d) The change of measured coefficient of friction (COF) with the increase of the normal force, which corresponds to the variation of deformation morphology in different deformation stages in (c). The critical force *F*_c_ indicates the initiation of crack. e) The critical force of crack initiation under scratch and the brittleness index for alloys R1 to R4.

In addition to plasticity, we also performed nanoscratch tests by nanoindentation under ramp mode (see **Figure** **4**a and Experimental Section) to investigate crack resistance of the alloys. We increase the normal force *F*_N_ under a constant rate while pulling the sample at a constant speed of *V*. This results in simultaneous increase of the lateral force *F*_µ_ (Figure 4b) and leads to deformation and fracture of surface materials under indenter. Figure 4c presents a representative scratch trace. The trace can be categorized into three different regimes corresponding to elastic deformation, plastic deformation, and fracture. The regimes can also be seen from the change of COF with *F_N_* (Figure 4d). There exists a critical normal force (*F*_c_) at which crack formation initiates under scratch. A larger *F*_c_ suggests higher crack resistance. Figure 4e shows the measured *F*_c_ values for the four alloys. As can be seen, the critical normal force of R1 (*F*_c_ = 7.5N) is significantly larger than any of the other three samples (≈1.1 N for R2, ≈0.9 N for R3 and ≈1.4 N for R4), indicating the substantially better crack resistance of R1. The better crack resistance of R1 than R2, R3 and R4 can be attributed to both chemical and structural effects. For MGs, their fracture toughness show strong compositional dependence and can vary from less than 10 MPa m^−1/2^ in some Fe‐based^[^ ^62^ ^]^ MGs to even 200 MPa m^−1/2^ in Pd‐based MGs.^[^ ^63^ ^]^ In addition, Ni is considered as harmful element to mechanical properties of MGs, since the addition of Ni into Zr‐based MGs can significantly deteriorate their fracture toughness.^[^ ^64^ ^]^ In here‐studied Ta–Ni–Ir MG forming system, the increase of Ni content also leads to the reduction of crack resistance (Figure 2c). NC metals are often reported to be less crack resistant due to the accumulation of defects at grain boundaries and triple junctions, which result in void formation and subsequent cracking.^[^ ^65^ ^]^ Therefore, the poor crack resistance of R4 with a NC structure is reasonable. To quantitatively compare the crack resistance of the four samples, we estimate brittleness index, (*H*/*E*_r_)(*H*/*K*_Ic_)^2^ proposed by Rhee et al.,^[^ ^66^ ^]^ which reflects the ratio between the critical load for yield (*F*_Y_) and fracture (*F*_F_) under indentation:

(1) $$F_{Y}/F_{F}=\left(D/A\right)\left(H/E_{r}\right){\left(H/K_{Ic}\right)}^{2}R$$

In the equation, *F*_Y_ can be calculated by *H* and *E*_r_ according to Hertzian elastic theory,^[^ ^66^ ^]^ while *F*_F_ of bulk materials can be estimated from *F*_c_ of thin films under scratch by considering both tangential load^[^ ^67^ ^]^ and thickness effects^[^ ^68^ ^]^ (see Supporting Information), *A* (≈8.6×10^3^) and *D* (≈0.85) are dimensionless parameters obtained by experimental data fitting,^[^ ^66^ ^]^ *K*_IC_ is the mode I fracture toughness, and *R* is the radius of contact indenter. *F*_Y_/*F*_F_ > 1 suggests brittleness while *F*_Y_/*F*_F_ < 1 suggests quasiplastic behavior. The estimated brittleness index of these four alloys is shown in Figure 4e. For R1, the brittleness index is ≈1900, which is two order smaller than the other three samples (≈10^5^) and most ceramics (>10^5^), indicating its strong crack resistance.^[^ ^66^ ^]^ The excellent crack resistance of R1 can be attributed to the profuse formation of shear bands (Figure S3, Supporting Information) which relieve strain localization and thus avoid early crack initiation.^[^ ^63^ ^]^ It is worth mentioning that the preliminary screening based on indent morphologies is consistent with the deformation behaviors observed with microcompression and scratch tests. In the past decades, plenty of attentions have been paid to the development of high‐throughput mechanical characterization methods for structural materials, in particular, the approach for rapid evaluation of plasticity and crack resistance.^[^ ^69^ ^]^ Our results demonstrate that indent morphology can be considered as an effective indicator in identifying MGs of better plasticity and crack resistance.

### 2.4 Tribological Properties of Selected Alloys

**Figure 5:**
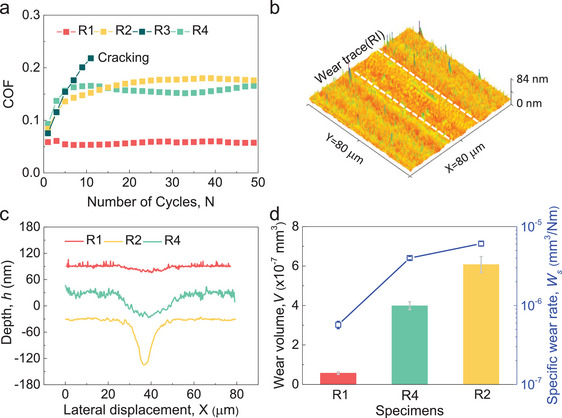
Friction and wear behavior of four typical Ta–Ni–Ir alloys from the combinatorial alloy library. a) The variation of coefficient of friction (COF) with the reciprocating lateral motion of indenter. b) The typical morphology of wear trace for R1. c) Cross‐sectional profiles of wear traces for alloy R1, R2, and R4. d) The wear volume and specific wear rate for alloy R1, R2, and R4.

With the combination of high strength, remarkable plasticity and crack resistance, the Ta‐rich MGs with high glass transition and crystallization temperature in R1 are expected to possess superior tribological properties. To verify this, spherical diamond ball (400‐µm‐diameter) is used to perform reciprocating sliding motion on each alloy in ambient air under contact force of 2 N (maximum Hertzian contact pressure of ≈5 GPa) with sliding velocity of 200 µm s^−1^ along a 1 mm stroke and repeated for 50 times until the friction coefficient is steady. As shown in **Figure** **5**a, R1 exhibits a steady state COF as low as ≈0.05, while that of the other three alloys are one order of magnitude higher, i.e., ≈0.17 for R2 and ≈0.15 for R4. The low COF of R1 can be attributed to the self‐lubrication of MGs with liquid‐like surface and its better structural stability owing to its better crack resistance than that of R2 and R4. We note that crack initiates in R3 after 10 times of cycling. This is consistent with the assertion deduced from nanoindentaion tests that the alloy is of poor crack resistance. In previous research, the friction of different metals, including both amorphous^[^ ^70^ ^]^ and crystalline^[^ ^71^, ^72^ ^]^ metals, has been tested by nanoindentation with diamond indenter. The COFs of several MGs^[^ ^70^ ^]^ are in the range of 0.1 to 0.2, while that of crystalline metals, typically for NC metals^[^ ^71^ ^]^ and HEAs,^[^ ^72^ ^]^ are larger than 0.2. It is clear that our Ta‐rich MG (R1) with COF of 0.05 shows outstanding friction behavior than other metals ever reported under diamond indenter and is comparable to that of diamond‐like materials.^[^ ^9^ ^]^

We further scanned the wear traces to estimate their wear volume (see Figure 5b,c and Figure S4, Supporting Information). A shallow and smooth wear track (see Figure 5b) is observed for R1 with wear volume of only ≈5.7 × 10^−8^ mm^3^. Although R2 is also of glassy structure, a blunt‐yet‐deep wear track occurs after sliding tests (see Figure S4a, Supporting Information). The wear volume is estimated to be 5.9 × 10^−7^ mm^3^ (Figure 5d), an order of magnitude larger than that for R1. As shown in Figure 3a, the yield strength of R2 (≈4 GPa) is only about 20% lower than that of R1 (≈5 GPa). However, the COF of R2 is more than three times higher than that of R1 and the wear volume of R2 is an order of magnitude larger than that of R1. Therefore, strength or hardness is not the only factor leading to the significant difference in COF and wear volume between R2 and R1. The main reason for the enhanced wear resistance in R1 can be attributed to its better crack resistance than R2. This is supported by the fact that R4 exhibits much higher yield strength (≈8 GPa) but deep and sharp wear trace. The wear volume for R4 is estimated to be 4.0 × 10^−7^ mm^3^, an order of magnitude larger than that for R1. This indicates that the tribological properties of MGs with good crack resistance can be better than crystalline metals even with high strength. We further estimated the specific wear rate, *W*_s_, defined as the removed volume per unit scratch distance per unit force. As shown in Figure 5d, *W*_s_ of R1 is only 5.7 × 10^−7^ mm^3^ N^‐^^1^m^−1^. The value is much lower than most conventional crystalline metals. Remarkably, this value is comparable to diamond‐like materials (10^−6^–10^−9^ mm^3^ N^‐^^1^m^−1^).

To further verify the diamond‐like wear behaviors of R1, we perform standard ball‐on‐disk wear tests with G‐Cr steel ball (4‐mm‐diameter) in ambient air with sliding velocity of 10 mm s^−1^, slide stroke of 5 mm and contact stress of ≈1 GPa (load of 5 N) for 3600 s (see Experimental Section). A steady state COF of ≈0.15 is obtained for R1, while that for R4 and R2 is ≈0.4 and ≈0.8, respectively (Figure S5a, Supporting Information). The morphology of wear surfaces (Figure S5b,c, Supporting Information) indicates that the wear track of R1 is narrow and smooth. The specific wear rate of R1 alloy is estimated to be 2.1 × 10^−7^ mm^3^ N^‐^^1^m^−1^, while that for R4 and R2 is 1.1 × 10^−6^ and 4.2 × 10^−6^ mm^3^ N^‐^^1^m^−1^, respectively (Figure S5d, Supporting Information). Since cracking occurs only after 10 min tests for R3, its specific wear rate is not estimated. Furthermore, we also characterized the rubbing surfaces of the Ta‐rich MG film and corresponding counterbodies of either diamond ball or G‐Cr steel ball (Figure S6, Supporting Information). It turned out that wear traces on both Ta‐rich films and their corresponding counterbodies are too shallow to be detected, especially for the wear trace under diamond ball. This strongly indicates the superior tribological properties of our Ta‐rich MG film. Chemical analysis by EDS (Figure S7, Supporting Information) confirms the chemical stability of the Ta‐rich film after 3600 seconds wear test. According to the results shown above, tribological properties of metals are not solely determined by hardness and strength but also enormously influenced by both plasticity and crack resistance that can be modified by chemical and structural variables. The high‐throughput method developed in this study provides an effective approach for discovering high performance alloys over a broad range of structure and composition.

**Figure 6:**
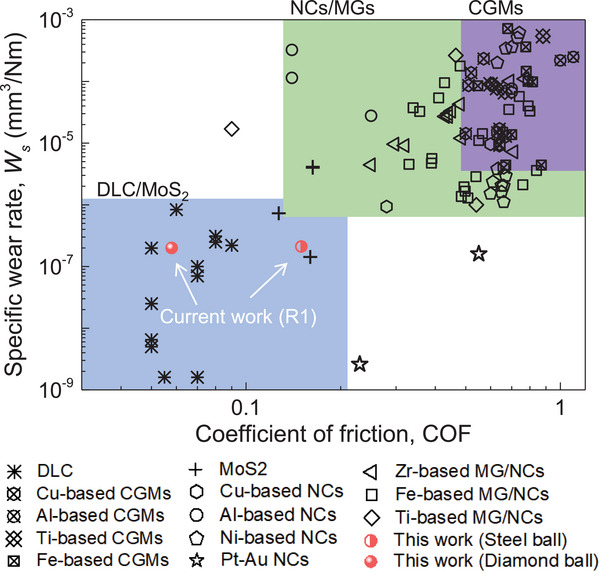
Summary of tribological properties of typical materials. The plot includes parameters for typical classes of materials that are developed for improved tribological properties, such as coarse‐grained metals (CGMs),^[^ ^14^, ^15^, ^16^, ^17^, ^23^, ^75^, ^76^, ^77^, ^78^, ^79^, ^80^, ^81^ ^]^ nanocrystalline (NC) metals,^[^ ^15^, ^16^, ^22^, ^23^, ^24^, ^74^, ^78^, ^82^, ^83^ ^]^ metallic glasses (MGs),^[^ ^17^, ^30^, ^74^, ^79^, ^80^, ^81^, ^84^, ^85^, ^86^, ^87^, ^88^, ^89^ ^]^ MoS_2_,^[^ ^90^ ^]^ and diamond‐like carbon (DLC).^[^ ^10^, ^73^, ^91^, ^92^, ^93^ ^]^

**Figure** **6** presents a summary of the friction and wear performance of representative materials, including conventional coarse grained metals (CGMs), typical MGs, the state‐of‐the‐art NC metals, DLC, and MoS_2_ etc. It is clear that both COFs and *W*_s_ of advanced metals, such as most MGs and NC metals are superior to conventional alloys, but are still inferior to carbon‐based materials, especially to DLC and MoS_2_ with COFs and *W_s_* lower than ≈0.2 and ≈10^−6^ mm^3^ N^‐^^1^m^−1^. By surface oxidation during wear, Liu et al.^[^ ^74^ ^]^ achieved a low COF of ≈0.09 in (TiNbZr)_75_Ag_25_ amorphous‐crystalline alloy. Despite its low COF, *W*_s_ of the alloy is still much higher than ≈10^−6^ mm^3^ N^‐^^1^m^−1^. Curry et al.^[^ ^24^ ^]^ achieved a low *W*_s_ of 10^−7^ or even 10^−9^ mm^3^ N^‐^^1^m^−1^ in a Pt–Au NC alloy. However, the alloy exhibits a high COF of 0.2 to 0.3 under Al_2_O_3_ ball or 0.5 to 0.6 under Si_3_N_4_ ball. These imply that it is challenging to achieve both ultralow COF and *W*_s_ in metals. In contrast, the Ta_62_Ir_21_Ni_17_ MG (R1) discovered in this study shows an ultralow COF of ≈0.05 under diamond ball (≈0.15 under steel ball) and specific wear rate of ≈10^−7^ mm^3^ N^‐^^1^m^−1^, both of which are comparable to carbon‐based materials. Despite of size limitation and environment sensitivity,^[^ ^9^, ^10^, ^11^ ^]^ the ultralow COFs and wear rates of carbon‐based materials make them good choice for plenty of applications, such as wear‐protective coatings for optics and magnetic storage media, tribological coatings for metal gears and bearings, and protective coatings for joint implants.^[^ ^73^ ^]^ With COFs and *W*_s_ comparable to diamond‐like materials, the application of high temperature of MGs can be greatly extended. On the other hand, high temperature MGs have been reported to be promising in a variety of technological applications at both ambient temperature and extreme conditions.^[^ ^29^ ^]^ For example, with excellent mechanical properties and thermoplastic formability, high temperature MGs are excellent candidates as mold materials for precision glass molding.^[^ ^32^ ^]^ The diamond‐like tribological properties of the Ta‐based MGs suggest that they are durable during glass molding. To reveal the superior thermal performances of our Ta‐rich high temperature MG, we performed the thermogravimetry analysis (TGA) and high temperature annealing to characterize its high temperature oxidation and crystallization resistance (Figure S8, Supporting Information). Substantial weight change does not occur until the temperature is beyond 920 K and crystallization cannot be detected even after annealing at 1073 K for 30 min. These results indicate that the Ta‐rich high temperature MGs possess superior thermal stability against structural changes. In addition, our results demonstrated that plasticity and crack resistance of the Ta‐rich MGs play vital roles in improving tribological properties. The high throughput mechanical characterization based on indent morphology can be an effective method for fast screening MGs with diamond‐like wear.

## 3 Conclusion

In conclusion, we successfully fabricated Ta‐rich MGs with diamond‐like tribological properties including both ultralow COF (≈0.05 for diamond ball and ≈0.15 for steel ball) and *W*_s_ (≈10^−7^ mm^3^ Nm^−1^). These superior tribological properties are attributed to the combination of enhanced structural stability, high strength, good plasticity, and superior crack resistance of the Ta‐rich MGs. To rapidly identify MGs with outstanding mechanical properties, we developed a high‐throughput method based on the different deformation responses of alloys. The differences of indent morphology due to the formation shear bands or cracks allow rapid identification of alloys with better plasticity and crack resistance. The strategy is simple but effective, which will be useful for developing high‐performance alloys aiming at industrial application.

## 4 Experimental Section

### Sample Fabrication

The combinatorial Ta–Ni–Ir thin film was fabricated by magnetron cosputtering deposition at a deposition rate of 8.8 nm min^−1^. Pure elements with purity better than 99.95% were used as sputtering targets. The film was deposited on 100‐mm‐diameter Si wafers. The base pressure of the chamber was higher than 1.0 × 10^−4^ Pa, and the working pressure was kept at 1.0 Pa by flowing high‐purity Ar gas. The deposited film had a thickness of 2 µm.

### Compositional and Structural Characterizations

The composition of combinatorial Ta–Ni–Ir thin film was analyzed by a Phenom scanning electron microscope (SEM) equipped with energy‐dispersive X‐ray spectroscopy (EDX). The structural characterizations were performed by XRD using a Malvern PANalytical Empyrean X‐ray diffractometer with a Cu‐K*α* radiation source covering a range of 20 to 65° with scan rate of 10° min^−1^. To achieve high‐throughput screening, a PIXcel^1D^ linear detector with 256 pixels in the detector array was used. The XRD mapping was realized by using a sample stage attached to a *x*–*y*–*z* triaxial motor. The characterizations were performed on a 19 × 19 matrix with a spacing of 5 mm.

### Mechanical Characterization

Mechanical tests were carried out on a Hysitron nanoindentaton system (Bruker Hysitron TI980). Reduced modulus and hardness was measured with partial unloading mode under low load with a Berkovich indenter and the maximum load for each cycle increases gradually from 100 to 8000 µN. The high‐throughput measurements were achieved by using the combi utility in TI980 by setting up a position groups with a 9 × 9 array with a spacing of 10 mm. Microcompression tests were conducted using a 10 µm flat indenter under displacement control up to 20% strain with strain rate of 5 × 10^−3^ S^−1^. Micropillars with a diameter of 1.2 µm were machined by FIB with their taper angle less than 2°. On the other hand, severe plastic deformation was introduced by Berkovich indenter under high load mode with displacement control. The indents were created to a depth of 2 µm within a time of 5 s. Nanoscratch (100 µm in diameter) and wear tests (400 µm in diameter) were conducted by employing scratch mode with nanoindentation. Nanoscratch tests were conducted under the ramp mode with the normal load increasing from 0 to 3, 5, and 10 N within 5 s. The sliding distance was 1 mm. Wear tests were performed under the constant mode with the normal load set to 2 N at room temperature and in ambient air (relative humidity of ≈50%), the reciprocating sliding motion of 50 times, a stroke length of 1 mm, and a sliding rate of 200 µm s^−1^. The standard ball‐on disk tests were performed on a UMT‐3 wear machine using a 4 mm G‐Cr steel ball with load of 5 N, sliding velocity of 10 mm s^−1^, an oscillating stroke of 5 mm, and a total sliding time of 60 min at room temperature and in ambient air (relative humidity of ≈50%). Wear tests under either nanoindentation or UMT‐3 wear machine were repeated at least three times.

### Morphology Characterization

The morphology observations were carried out by using SEM. The wear traces were characterized by nanoindentation (Bruker, TI980) under mapping mode, and 3D‐optical profiler (Bruker, ContourX‐200).

### Thermal Performance Characterization

The high temperature oxidation resistance was performed by TGA in a TGA/SDTA851e instrument. The Ta‐rich MG films were grind into powders with weight of 12.26 g and scanned from 323 to 1273 K in air at a constant heating rate of 0.333 K s^−1^. This work also annealed the Ta‐rich MG film (on Si substrate) at 1073 K for 30 min in high vacuum (3 × 10^−5^ Pa) and then characterized its structure with XRD with a Cu‐K*α* radiation source covering a range of 20° to 100°.

## Conflict of Interest

The authors declare no conflict of interest.

## Author contributions

Y.H.L conceived the idea and supervised the project. F.C.L and M.X.L. performed the combinatorial syntheses, chemical analyses, and structural characterizations. F.C.L. performed mechanical, tribological, morphological characterizations. F.C.L. and Y.H.L analyzed the data with contribution of M.X.L., W.C., S.Y.T., and W.W.H.. F.C.L. and Y.H.L. wrote the manuscript with input from all authors.

## Supporting information

Supporting Information

## Data Availability

The authors declare that the data supporting the findings of this study are included within the paper and its Supplementary Information file.
