# Supplementary material for: Achieving Diamond‐Like Wear in Ta‐Rich Metallic Glasses
Source: Adv Sci (Weinh). 2023 May 21;10(22):2301053. doi: 10.1002/advs.202301053 (PMC10401100; doi:10.1002/advs.202301053)
Supplement: Supplementary file 1 — Supporting Information [file ADVS-10-2301053-s001.pdf]

ADVANCED SCIENCE
Open Access

Supporting Information

for *Adv. Sci.*, DOI 10.1002/advs.202301053

Achieving Diamond-Like Wear in Ta-Rich Metallic Glasses

*Fucheng Li, Mingxing Li, Liwei Hu, Jiashu Cao, Chao Wang, Yitao Sun, Weihua Wang and Yanhui Liu**

Supporting Information for

Achieving diamond-like wear in Ta-rich metallic glasses

Fucheng Li [1, 2], Mingxing Li [1], Chao Wang [1],Yitao Sun [1], Weihua Wang [1, 2, 3],
Yanhui Liu [1, 2, 3*]

[1.] Institute of Physics, Chinese Academy of Sciences, Beijing 100190, China

[2.] Songshan Lake Materials Laboratory, Dongguan 523808, China

[3]Central of Materials Science and Optoelectronics Engineering, University of Chinese Academy of Sciences, Beijing, China.

[*] Correspondence to: yanhui.liu@iphy.ac.cn

To estimate F_F of bulk materials under indentation from F_c of thin films under scratch, both tangential load [1] and thickness effects [2] need to be taken into consideration. Firstly, the critical normal load, F_c for the initiation of cracking of thin films under scratch can be related to the critical load, F_n for fracture under frictionless conditions as following [1]:

$$F_n = (1 + k\mu)^3 F_c \quad (1)$$

Where $k = \frac{3\pi}{8}\frac{4+\nu}{1-2\nu}$ and ν is poisson's ratio with value of 0.3, and μ the friction coefficient. Secondly, Lee et al. [2] systematically investigated the thickness effect on the critical conditions for radial cracking in thin coatings (d<1 mm). They found that there is a critical thickness d_c, before which, the critical load F_n for the formation of radical cracking in thin films is proportional to squared thickness ($F_n \propto d^2$). When the thickness is larger than d_c, the substrate effect can be eliminated and the critical load F_n becomes insensitive to film thickness and is equivalent to F_F. Here, we propose that the critical thickness d_c, should be at least larger than the size of plastic deformation zone (2.4a), where a is the contact radius and can be estimated by Hertzain theory with $\mathrm{a} = \left(\frac{3\mathrm{F_n R}}{4\mathrm{E_r}}\right)^{1/3}$. Taking the contact depth, h into consideration, d_c should be larger than 2.4a+h. Therefore, the relationship between Fn and F_F can be built as following:

$$F_F \geq \left(\frac{2.4a+h}{d}\right)^2 F_n \quad (2)$$

Combining Eq. (1) with Eq. (2), we can build the correlations between F_F and F_c as following:

$$F_F \geq (1 + k\mu)^3 \left(\frac{2.4a+h}{d}\right)^2 F_c \quad (3)$$

References

1 Rhee, Y.-W. et al. Brittle fracture versus quasi plasticity in ceramics: a simple prdeictive index. J. Am. Ceram. Soc. **84**, 561-565 (1969).

2 Gilroy, D. R. & Hirst, W. Brittle fracture of glass under normal and sliding loads. J. Phys. D: Appl. Phys. **2**, 1784 (2001).

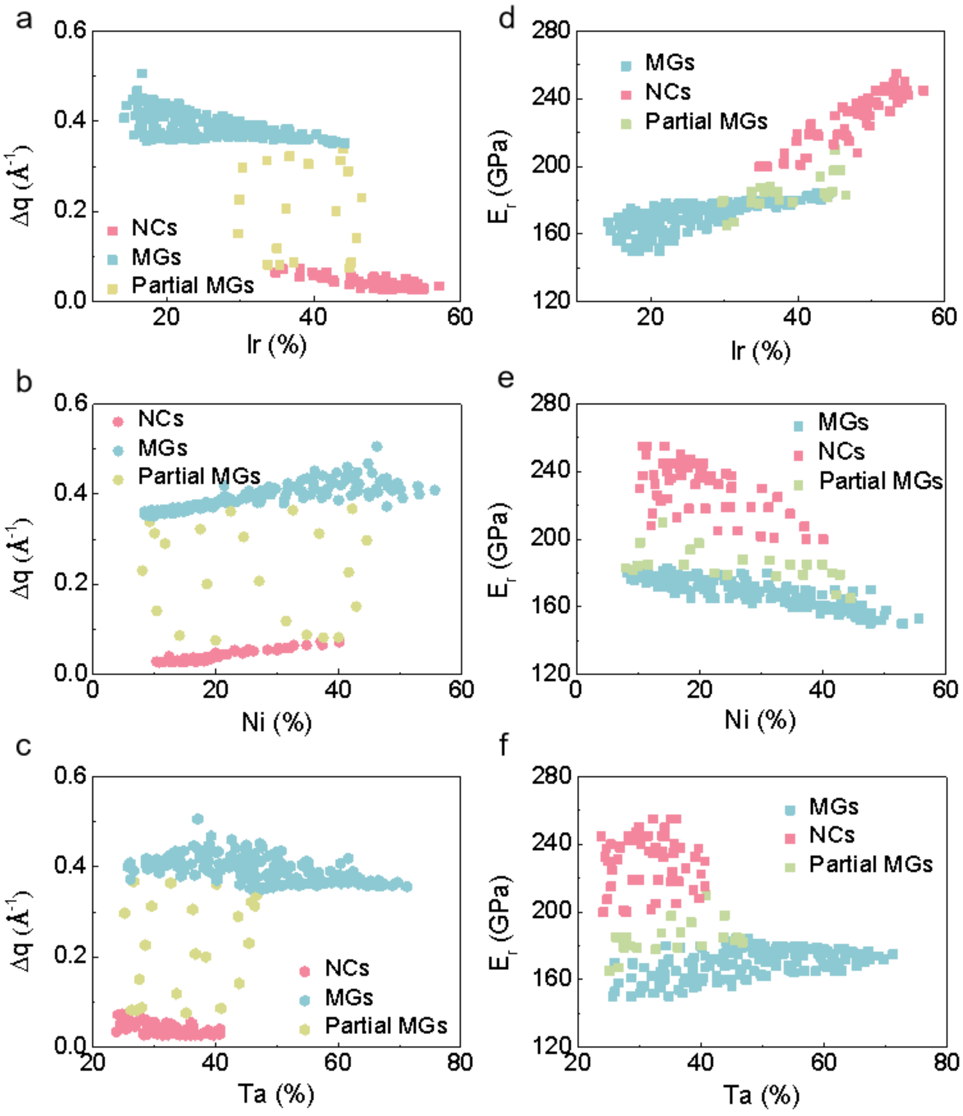

Supplementary Figure 1 Evolution of structures and mechanical properties with varying Ir(%), Ni(%) and Ta(%). The evolution of Δq with elements **a.** Ir (%), **b.** Ni (%) and **c.** Ta (%.), The evolution of reduced elastic modulus with elements **d.** Ir (%), **e.** Ni (%) and **f.** Ta (%).

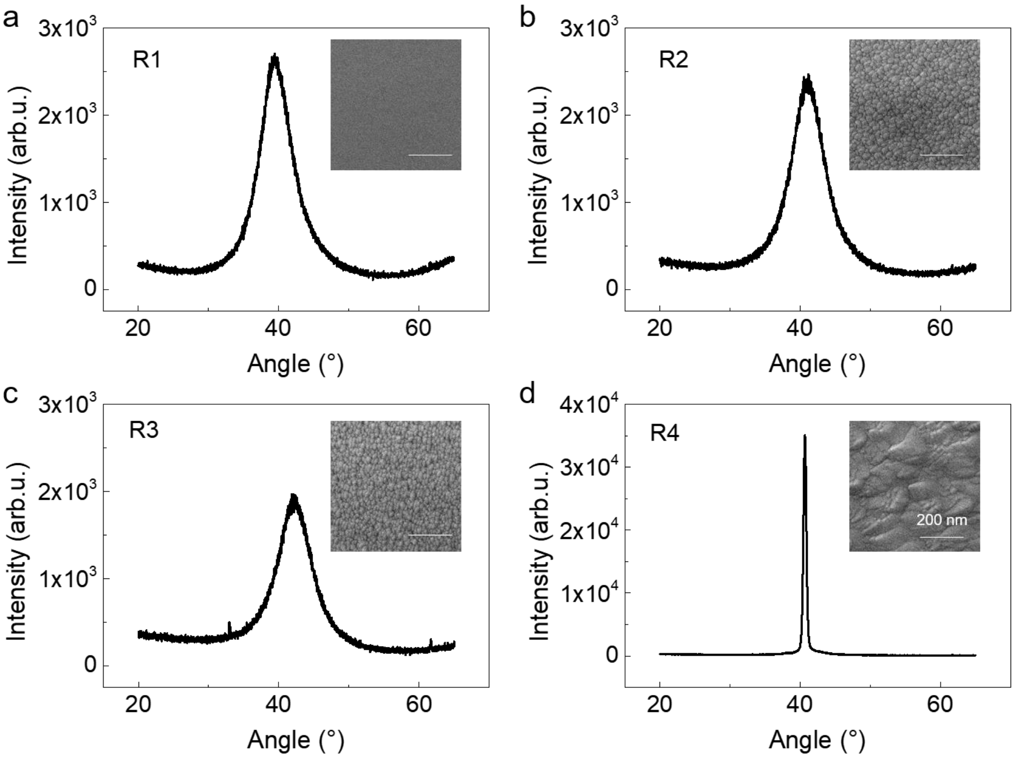

Supplementary Figure 2 The XRD patterns and surface morphologies of samples

a. R1**, b.** R2**, c.** R3 **and d.** R4**.**

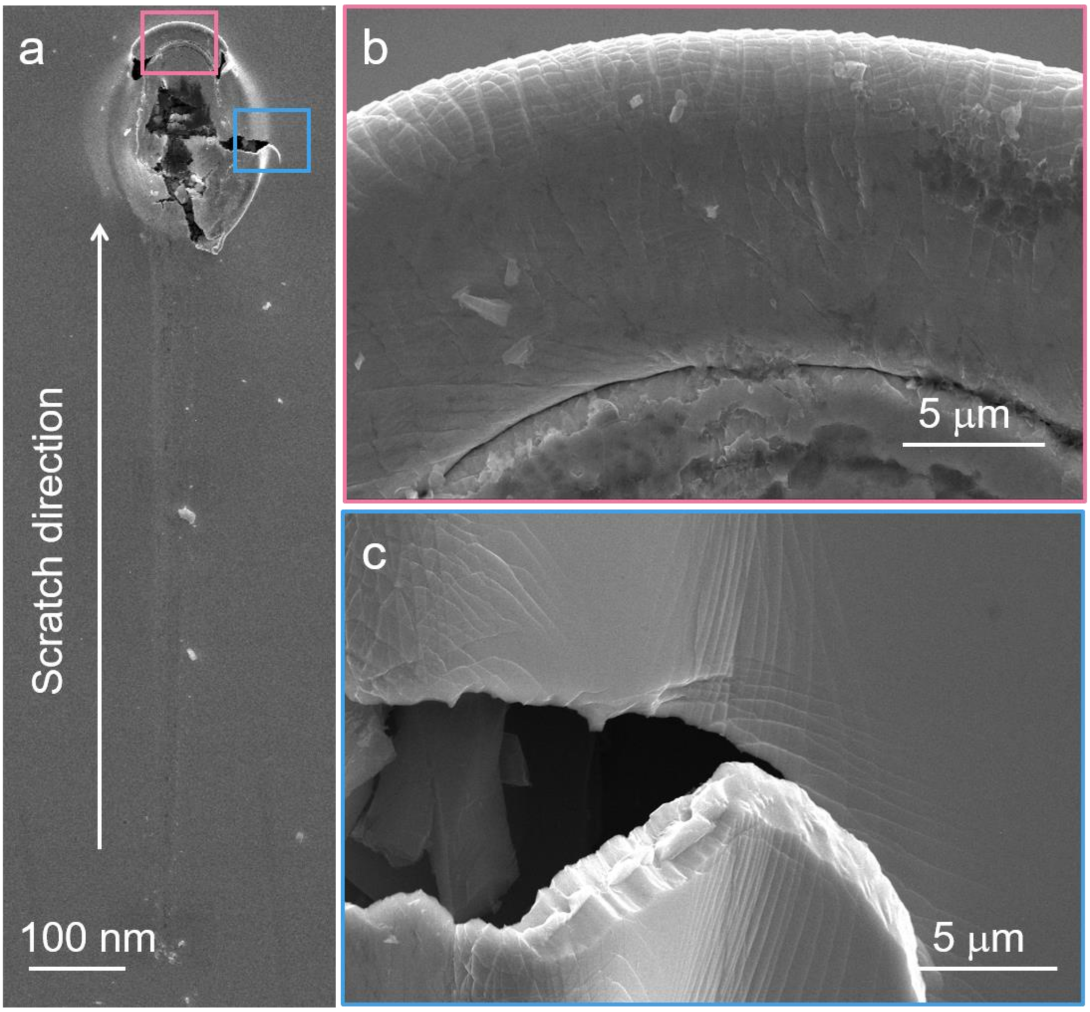

Supplementary Figure 3 The scratch morphology of R1 sample under ramp mode with normal force up to 10 N. a. the scratch trace of R1 sample, **b** and **c.** plenty of shear bands around the crack tips.

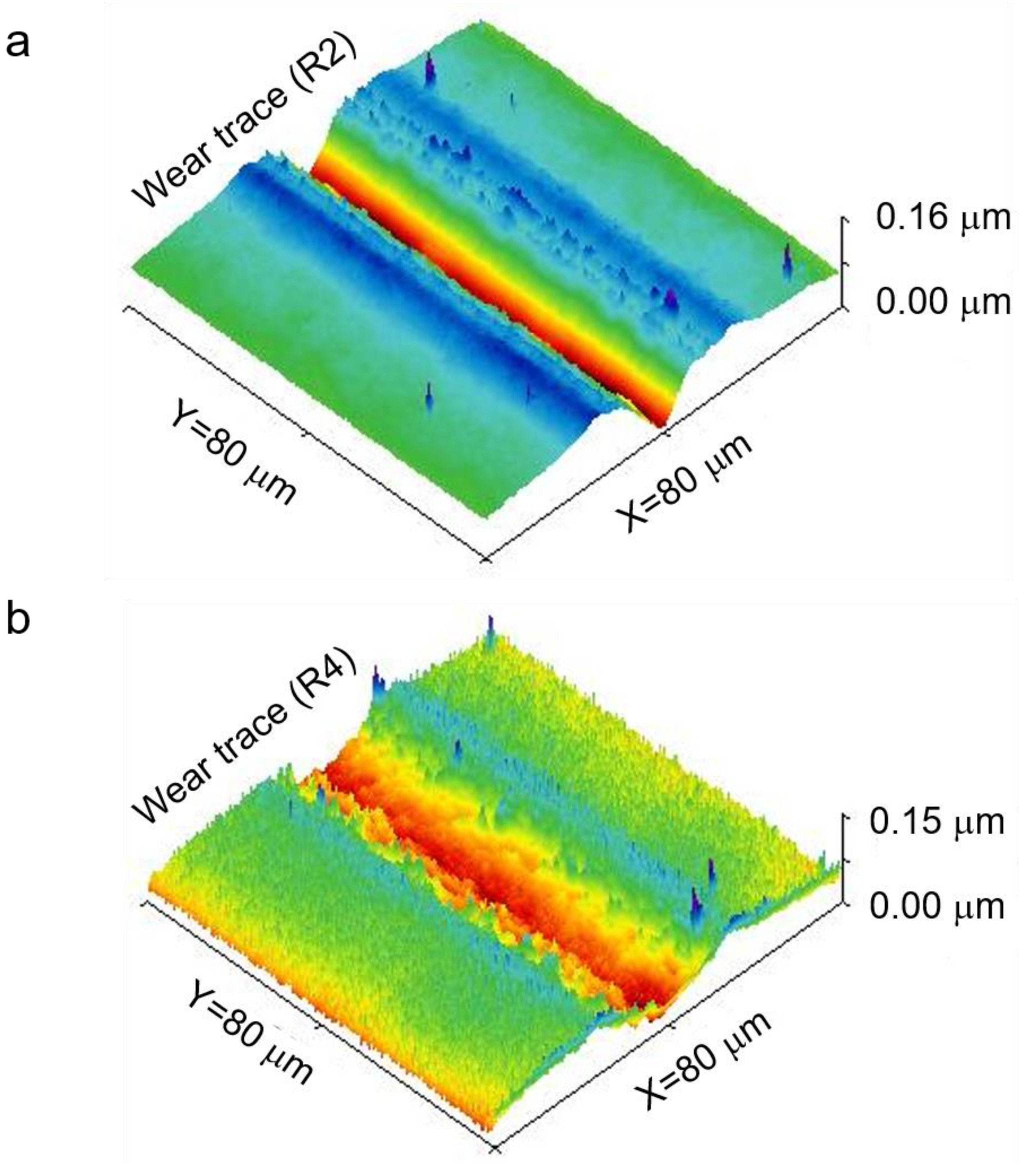

Supplementary Figure 4 The typical morphologies of wear trace for a. R 2 **and b.** R4

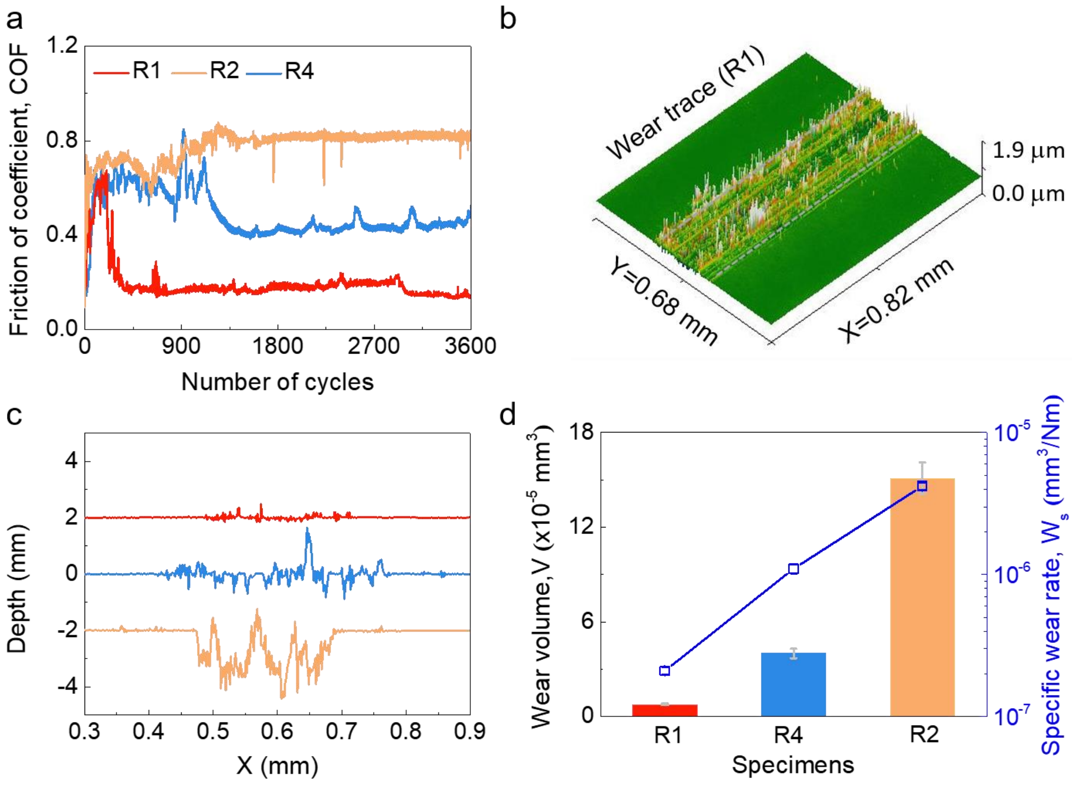

Supplementary Figure 5 Standard ball-on-disk tests with G-Cr steel ball on Ta-Ni-Ir films. a. Friction of coefficient for $Ta_{62}Ni_{17}Ir_{21}$ (R1), $Ta_{39}Ir_{33}Ni_{28}$ (R2) and $Ir_{50}Ni_{19}Ta_{31}$ (R4) films, b. 2D cross-sectional profiles of wear traces for R1, R2 and R4, c. The typical 3-D morphology of wear trace for R1, d. The summary of wear volume and specific wear rate for R1, R2 and R4.

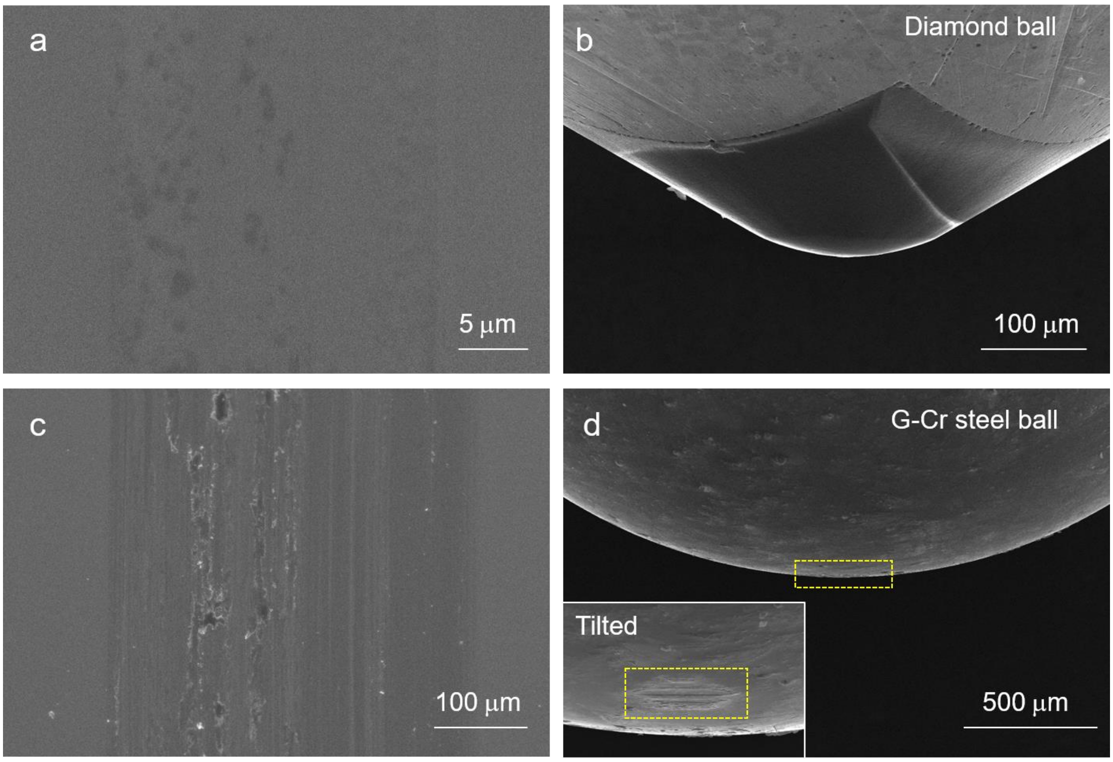

Supplementary Figure 6 The characterization of rubbing surfaces of both the Ta-rich MG film and corresponding counterbodies with SEM. a. The rubbing surface of the Ta-rich MG under the wear of diamond ball, b.The rubbing surface of diamond ball after wear tests, c. The rubbing surface of the Ta-rich MG under the wear of G-Cr steel ball, b.The rubbing surface of G-Cr steel ball after wear tests, inserted with its tilted image to show the wear trace on steel ball.

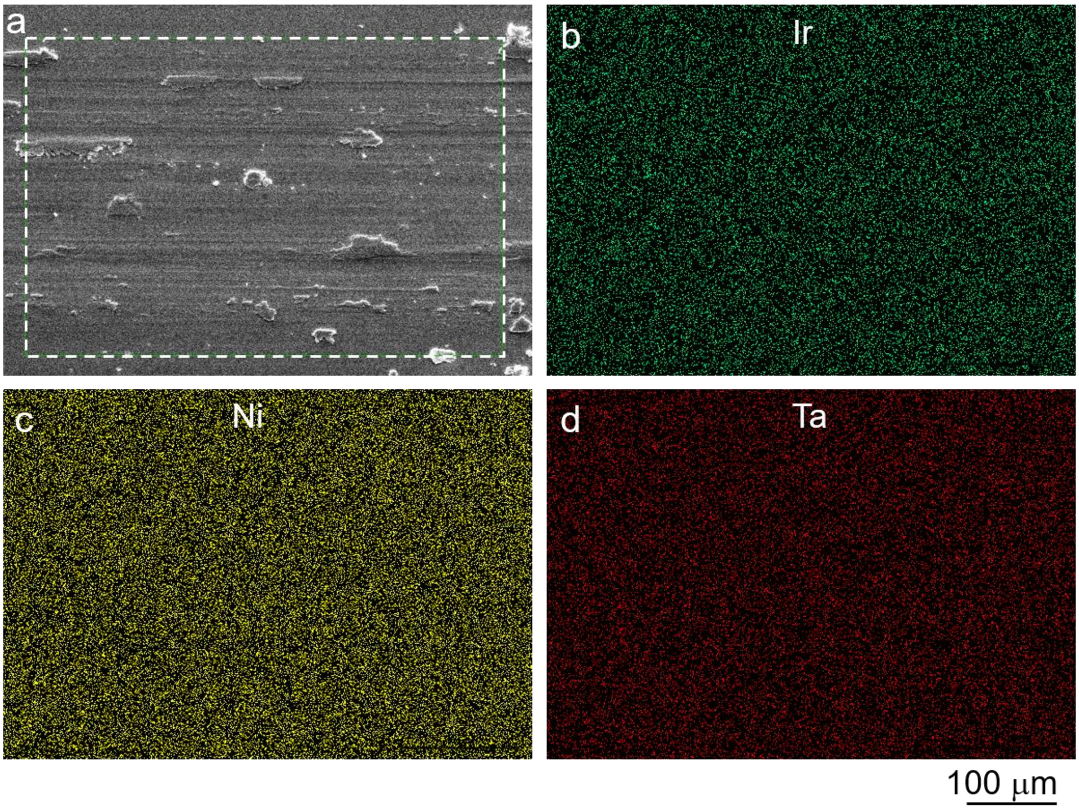

Supplementary Figure 7 The chemical characterization of the rubbing surface of the Ta-rich MG film with EDS. a. The wear trace of R1, b. The chemical distribution of Ir element, c. The chemical distribution of Ni element, d. The chemical distribution of Ta element.

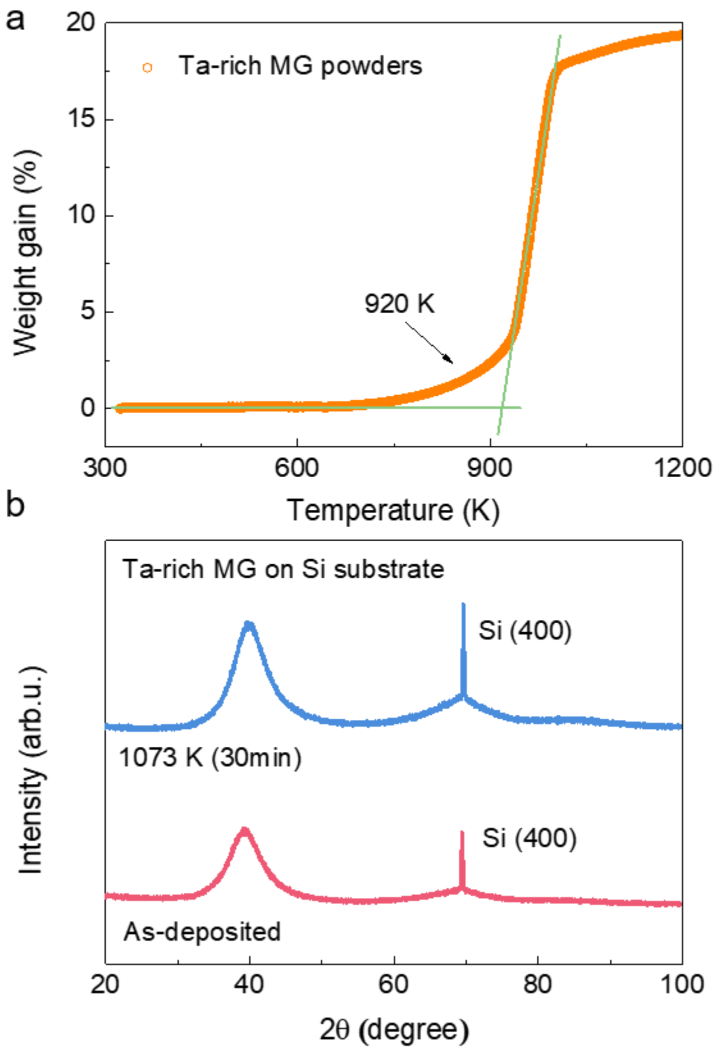

Supplementary Figure 8 The thermal performances of the Ta-rich MG. a. the thermogravimetry analysis of the Ta-rich MG powders, b. the XRD result of the as-deposited and 1073 K annealed (30min) Ta-rich MG on Si substrate.
